# Medico-Chirurgical Transactions. (2nd Notice)

**Published:** 1846-04

**Authors:** 


### Medico-Chirurgical Transactions, Published by the Royal Medical and Chirurgical Society of London. Volume the Twenty-eighth (the Tenth of the Second Series) 1845.

[Continued from page 216.]

### VIII. Observations on Cleft Palate, and on Staphyloraphy. By *W. Fergusson,* Esq. Professor of Surgery in King's College, London.

The author, after making some remarks on the condition of the parts in this malformation, briefly notices the various operative proceedings which have been adopted to remedy the inconvenience, and particularly the incisions which have been made by operators with the intention of facilitating the approximation of the edges of the fissure. Mr. Fergusson calls attention to certain conspicuous movements in the soft flaps which may be noticed during the examination of the throat of a person whose palate is cleft. The flaps may be seen under four different conditions. " First. If the parts be not irritated in any way, the gap will be quite conspicuous, the lateral flaps will be distinct, and the posterior nares, with the upper end of the pharynx, will be observed, above and behind. Second. If the flaps be touched, they will in all probability be jerked upwards by a motion seemingly commencing at the middle of each. Third. If the parts be further irritated, as by pushing the finger against them into the fissure, each flap is forcibly drawn upwards and outwards, and can scarcely be distinguished from the rest of the parts, forming the sides of the nostrils and throat. And, fourth. If the parts further back be irritated, as in the second act of deglutition, the margins of the fissure are forced together, by the action of the superior constrictor muscle."

All these conditions and movements are, in the author's opinion, very readily accounted for. In the first instance, the parts may be deemed in a quiescent state; in the second, the levatores palati are called into play, and move the flaps as described; and in the third, these muscles act still more forcibly, and the palato-pharyngei will join in drawing the parts outwards. The fourth condition need not be again described.

If the free margin on one side of the fissure be seized with the forceps, drawn towards the mesial line, and the flap be then irritated, it will be drawn upwards and outwards with remarkable force; this movement, it is evident to the author, can only be effected by two muscles, the levator palati and palato-pharyngeus. These muscles, then, he regards as the chief mechanical obstacles to the junction of the margins in the mesial line. He considers that the tensor palati and palato-glossus have but little influence in widening the fissure. Mr. Fergusson therefore proposes, as an important accessory to the operation of staphyloraphy, that the surgeon should, on strictly scientific grounds and in accordance with the modern principles of myotomy, so conduct his incisions as to destroy all motory power in the soft palate for the time being, and thus permit that repose of the stretched velum which is so essential to a happy result; in other words, he advises the division of the levator palati, the palato-pharyngeus and the palato-glossus muscles. The first of these he deems of the greatest importance, the second scarcely less so, and the third may be effected or not, as the circumstances seem to demand. Within the last twelve months he has operated on two cases of congenital cleft palate, in accordance with these views, and the results have been most satisfactory, as regards the union of the lateral portions. The steps which he follows are these:

" With a knife whose blade is somewhat like the point of a lancet, the cutting edge being about a quarter of an inch in extent, and the flat surface being bent semicircularly, I make an incision about half an inch long, on each side of the posterior nares, a little above and parallel with the palatine flaps, and across a line straight downwards from the lower opening of the Eustachian tube, by which I divide the levator palati muscle on both sides, just above its attachment to the palate. Next I pare the edges of the fissure with a straight blunt-pointed bistoury, removing little more than the mucous membrane; then, with a pair of long blunt-pointed curved scissors, I divide the posterior pillar of the fauces, immediately behind the tonsil, and, if it seems necessary, cut across the anterior pillar too; the wound in each part being about a quarter of an inch in extent. Lastly, the stitches are introduced by means of a curved needle, set in a handle; and, the threads being tied so as to keep the cut edges of the fissure accurately in contact, the operation is completed. These different incisions may be made in the order here detailed, or possibly it may be found most convenient to divide the palato-pharyngeus first, next the levator palati, and then to pare the edges when the muscular action has been taken off.

"Each of these steps requires some little separate notice. The first incision, it will be remarked, differs from all others hitherto proposed, and is founded on consideration of the anatomy of the parts. The levator palati, I have no doubt, is the main obstacle to the approximation of the margins, and is the principal cause of unsteadiness in the velum during the operation and after it has been accomplished. Its division may be effected through the method above recommended, but should the flap appear tense after the knife has been used, the incision may be further extended in case the muscle may not have been completely cut across. The extension of the incision, even without reference to the division of muscular fibres, will aid greatly in relaxing the sides of the palate. In many instances I believe that the levator muscle might be divided by a submucous incision, by plunging the blade through the mucous covering, and then moving it freely across the muscle." P. 292.

The parts concerned in the operation are represented in a woodcut, which is not, however, calculated to be of much assistance in guiding the surgeon. We need not follow the author in his minute description of the various manipulations and modes of overcoming the difficulties of this operation. Those who may wish to test the plan recommended will no doubt consult the paper itself. There is an Appendix giving an account of the two cases, referred to in this paper.

The suggestions of Mr. Fergusson are ingenious and merit consideration, but we must confess that we are not sanguine as to their results in diminishing the number of failures which unfortunately are of frequent occurrence after the operation of Staphyloraphy by the most skilful surgeons.

### IX. On the Pulsating Tumours of Bone, with the Account of a Case in which a Ligature was placed around the Common Iliac Artery. By *Edward Stanley*, F.R.S.

The recent occurrence of a case in St. Bartholomew's Hospital of pulsating tumour originating in the walls of the pelvis, in which a ligature was placed around the common iliac artery, has induced Mr. Stanley to place before the Society, in connection with the narrative of this case, those circumstances in the history of the tumours of bone which bear on the important fact, that certain of these tumours possess a pulsation identical in character with the pulsation of aneurism. He states that, three distinct sources of pulsation in tumours can be recognised. 1. The proximity of a large arterial trunk. 2. The development of blood-vessels and blood-cells constituting a sort of erectile tissue within the tumour. 3. Enlargement of the arteries of the bone in which the tumour has originated.

*Of pulsation dependent on the proximity of a large arterial trunk,* several cases are related. In one, a tumour of the arm, the ligature of the subclavian artery was recommended but not assented to. In another, in which there was a painful swelling above the knee and around the back part and sides of the lower-third of the thigh, it was agreed in consultation that sufficient ground existed for believing the tumour to be a popliteal aneurism, and accordingly the femoral artery was tied. Four other cases are mentioned, and Mr. Stanley remarks :—

"Here are six examples of pulsating tumours differing in their nature, and originating in different bones, but agreeing in the circumstance that no other source of pulsation was discoverable in them, than the contiguity of large arterial trunks; and to the same class of cases, the important one recorded by Mr. Guthrie appears to belong, in which a tumour about as large as an adult head, situated upon the right nates of a female, presented so decidedly the characters of aneurism, that it was believed to be so by Sir Astley Cooper, and other experienced surgeons who were consulted upon the case, and, accordingly, a ligature was placed around the common iliac artery." P. 308.

*Of pulsation dependent on the development of blood-vessels and blood-cells constituting a sort of erectile tissue within it.*—The case on which this paper is founded appears to have been an example.—The author describes another case of pulsating tumour originating in the femur.

*Of pulsation dependent on the enlargement of the arteries of the osseous tissue.*—A few cases which have been recorded are briefly alluded to. The author adduces a case of tumour of the thigh-bone, communicated to him by Mr. Luke, who, supposing it to be an aneurism, tied the femoral artery. On examining the limb, the lower-third of the femur was found expanded into a spherical tumour, in the interior of which were cells of varying size, some of the largest about an inch in diameter, and filled with blood.

Mr. Stanley remarks—

"There is one circumstance in the history of these pulsating tumours which requires especial notice, as it appears to have a material influence on the production of pulsation in them: this is the density and resistance of the structures which immediately invest them. In the instances of the development of the tumour within a bone, a thin osseous shell with thickened periosteum is its first and immediate investment; and in the instances of the development of the tumour upon the outer surface of a bone, thickened periosteum alone will be its first investment: moreover, according to the situation which the tumour happens to occupy, it will be more or less closely confined by the muscles immediately around it, which may then add considerably to the density and resistance of its coverings. Such were the circumstances of the case, recently in St. Bartholomew's Hospital, wherein the pulsating tumour, growing from both surfaces of the ilium, was in great part covered by the periosteum, much thickened, and it was, besides, closely confined by the iliacus internus muscle stretched over it on one side, and by the glutæus medius on the other. It may indeed be doubted whether any of these tumours would pulsate without the resistance derived in one or other direction from the bone or its coverings." P. 313.

In one of the cases above alluded to, a medullary tumour developed in the head of the tibia pulsated in its early stage, but ceased to pulsate directly it had burst through the resistant coverings of the bone. A tumour originating in soft parts, unconnected with any bone, but situated close to a large artery, and confined within existing structures, and thus approximating in its conditions to the pulsating tumour of bone, may, like it, pulsate in a manner to be mistaken for aneurism, of which Mr. Stanley gives an example. A patient under the care of Mr. Earle had a pulsating tumour below the left clavicle, and accordingly a ligature was placed around the subclavian artery. Some years afterwards it was ascertained that the tumour had originated within the sheath of one of the axillary nerves, and had derived its pulsations from the artery.

After some observations, tending to show the little value to be attached to the existence of a bellows-sound in the diagnosis between aneurism and the pulsating tumour of bone, Mr. Stanley proceeds to relate the case of pulsating tumour of the ilium, wherein a ligature was placed around the common iliac artery. Our space does not admit of our entering upon the details of this case, without which the reader would be unable to appreciate the difficulties of the diagnosis. It appears that, in consultation, the preponderance of opinion was in favor of the tumour being an aneurism, and the operation was accordingly performed. The patient afterwards died of peritonitis. The tumour was composed of a spongy tissue with cells and convoluted vessels distributed through it. There was a tumour on the inner side of the right arm identical in structure with that of the tumour of the pelvis.

In a postscript, Mr. Stanley refers to some similar cases contained in a memoir on pulsating tumours, designated aneurisms of bone, lately presented to the Royal Academy of Medicine in Paris by M. Roux. In a case of tumour originating in the lower end of the radius, the brachial artery was tied. In another case of tumour projecting from the head of the tibia the femoral artery was tied, after which the swelling disappeared. These cases are regarded as examples of pulsating tumour of bone, wherein blood-vessels, constituting a sort of erectile tissue, are developed within the tumour.

The particulars are added, of two other cases of pulsating tumour, originating in bone, in which the main artery of the limb was tied, on the supposition that the disease was aneurism.

This is a paper of considerable interest and practical value, as showing the necessity for great care in examining deep-seated pulsating tumours and caution in deciding upon their nature. The many cases of error in diagnosis leading to mistaken practice, which Mr. Stanley has taken the pains to collect in addition to his own, do not constitute a very flattering commentary on the state of surgical science.

### X. Description of a Malformation of the Duodenum, with Notices of analogous Cases. By *Robert Boyd*, M.D., Resident Physician St. Marylebone Infirmary.

The malformation described in this paper was found on examination of the body of a male still-born infant. The duodenum was much enlarged, and appeared like a bladder two-thirds filled, and contained a greenish-coloured fluid; the lower or most distant part from the stomach was imperforate and of larger calibre than the upper part. At its lowest part the duodenum was closed by a transparent membrane. Two inches and a quarter above this, a valve extended across, nearly half closing the gut. The great intestines were unusually small. The author mentions several similar cases described by other writers.

XI. Case of remarkable Hypertrophy of the Fingers in a Girl, with a Notice of some similar Cases. By *T. B. Curling,* Lecturer on Surgery, &c. London Hospital.

The subject of this curious congenital malformation was Eliza Hitchcock, a young woman aged 15, an inhabitant of Bethnal Green. The following is an account of their present state, which is represented in two excellent plates.

"On the right hand, the fore, middle, and ring fingers are of unusual size. The relative enlargement of the fore and ring fingers is only slight, but the middle one is of extraordinary proportions. It measures as much as five inches and a half in length, and four in circumference of the first phalanx, and is in every respect properly formed. On the left hand, the thumb, index, and middle fingers are hypertrophied, the ring and little fingers remaining of the natural size, and presenting a curious appearance, in contrast with their gigantic neighbours. The finger most enlarged is the index, which measures five inches and a quarter in length, and four inches in circumference. The thumb and middle finger are hypertrophied in a less degree. The middle finger has a lateral incurvation outwards, occasioned apparently by a displacement of the extensor tendon, which forms a bridle along its outer edge. The other enlarged fingers are of the normal shape.

"All parts of these hypertrophied fingers are equally developed in excess—the bones, articulations, integuments, and nails. The middle finger of the right hand and index finger of the left, which have attained the greatest growth, are fixed in an extended position, the girl being unable to bend them. The motions, however, of the several articulations are not destroyed, but the fingers are stiff, probably from long continuance in the straight position; and the girl's inability to bend them appears to be owing to the flexor muscles in the fore-arm not having acquired a development corresponding to the fingers upon which they act, as the motions of those fingers which are hypertrophied in a less degree, are very little, if at all, impaired. The other parts of the upper extremities are in proper proportion to the size of the body. There is merely a fulness at those parts of the hands from which the hypertrophied fingers proceed. Indeed, with these exceptions, all the different parts of the body are of appropriate size and form." P. 339.

The fingers in which the hypertrophy is most remarkable, are only half an inch shorter than the corresponding fingers of the famous giant O'Byrne.

To this case the author adds the particulars of one communicated to him by Professor Owen, and those of another remarkable case for which he is indebted to Mr. Paget. Mr. Curling also gives an account of a cast of a hand thus deformed which is contained in the Museum of King's College, London, and notices two published cases.

In four of the foregoing cases, the hypertrophied fingers were bent to one side, and the author suspects that, in all of them, this inclination was produced, as in the case of Eliza Hitchcock, by the tension of the displaced extensor tendon, which had not elongated in proportion to the increase in the size of the finger. In reference to the treatment of this disgusting deformity, Mr. Curling recommends, in a case where one finger only was enlarged to a great extent, and at the same time nearly useless, and interfering with the motions of the adjoining fingers, the removal of the hypertrophied part. In other cases, the enlarged finger might be reduced to a more moderate size by amputation of the distal phalanx.

### XII. On the Ophthalmia of Puerperal Women. By *Robert Lee*, M.D., F.R.S.

The author commences this paper by noticing the observations made by previous writers on this affection. He quotes a case recorded by himself in the 15th volume of the Society's Transactions, and another which occurred in 1832, as tending to prove that a relation exists between uterine phlebitis and the ophthalmia of puerperal women. Many cases of uterine and crural phlebitis came under his observation, during the twelve years which intervened between 1832 and 1844; but in none of these did inflammation of the eyes take place, and an opportunity present itself of proving by dissection, that the ophthalmia of puerperal women is the consequence of inflammation and suppuration of the uterine veins, or that there is a close relation between them. In the month of October, 1844, a case occurred which appeared to establish, in the most conclusive manner, the truth of this view of the pathology of this affection. The point alluded to we consider to be already quite established by the observations of other pathologists, as well as by those of Dr. Lee. We need not therefore dwell on the case before us, which is however a good example of this form of ophthalmia.

### XIII. Additional Observations on Obstructions of the Pulmonary Arteries. By *James Paget*, F.R.C.S., Lecturer on Physiology, and Warden of the College at St. Bartholomew's Hospital.

This case is intended as an appendix to the paper on the same subject in the last volume of these Transactions. It will be recollected that, in our April number (1845), we gave rather a full account of Mr. Paget's previous observations. The present paper contains an interesting case bearing upon the subject of coagulations in the pulmonary arteries. The author's remarks on the effects of these coagulations in the circulation, and functions of the lungs, cannot well be condensed, but deserve to be carefully read by pathologists. In conclusion, he ventures "to suggest that many cases of sudden death, for which no cause has been found, or which have been ascribed to some insufficient or improbable cause, have depended on clots obstructing the pulmonary arteries; and that the clots of the same kind, which are often found in the systemic veins, and are usually ascribed to phlebitis, though the coats of the veins are healthy, are the consequence of stoppage of the blood similar to this which I have described, and from a similar cause. It is no argument against the supposition of the frequency of such arrests or slow movements of the blood that the pulse continues: for the pulse is not, necessarily, an indication that the blood is flowing through the vessels, any more than a wave is sure evidence of a tide."

In a postcript the author briefly notices the observations of Dr. Angelo Dubini on the same subject, published in the Annali Universali di Medicina.

XIV. Two Cases of Anæsthesia and Loss of Motory Function of the Fifth Nerve. By *James Dixon,* Esq. Assistant Surgeon to the Royal London Ophthalmic Hospital.

The author remarks that an especial interest is imparted to the diseases of the fifth cerebral nerve, by the intimate connection which subsists between it and the senses of sight, smell, and taste: and as the very mechanism of one of these senses, namely, taste, is still a subject of dispute amongst physiologists, he trusts that a report of two cases of anæsthesia of the fifth nerve, which have lately been under his notice, may prove interesting to the Society. In one case, the eye on the affected side became inflamed; and vision was destroyed by a deposit of lymph in the pupil, anterior chamber and substance of the cornea; the same changes taking place in the eye, as follow the division of the 5th nerve in Magendie's vivisections. For the sake of comparison, the author arranges the results of Magendie's experiments and the symptoms observed in this patient's case in opposite columns; and it appears that they correspond in every particular, except as regards the state of the eye and vision.

In the second case, complete anæthesia had existed for almost a year and a half; and yet there was not the slightest inflammation of the eye, nor any opacity of its humors. In this latter patient, careful experiments were made, to ascertain what effect had been produced upon the tongue in respect of feeling and taste. Both were perfect on the right side. On the left, all the anterior part of the tongue on the left side was deprived of taste and feeling, while both these senses were unimpaired in that portion of the organ to which the lingual branch of the glosso-pharyngeal nerve is distributed.

In a note appended to this interesting communication, Mr. Dixon notices the much greater frequency of disease affecting the fifth nerve on the left, than on the right side of the body. Out of 47 recorded cases of the kind, 30 occurred on the left side, and only 12 on the right; whilst, in five patients, both nerves were affected at the same time.

XV. Account of a Case of External and Internal Cephalhæmatoma complicated with Fracture of the Right Parietal Bone, in a New-born Infant. By *Charles West,* M.D. Lecturer on Midwifery at the Middlesex Hospital, &c.

In this case, a small swelling, the size of a walnut, was noticed a little to the right of the vertex on the third day after birth. The swelling increased in size until it formed a soft elastic non-pulsating tumour of irregularly oval outline, occupying nearly the whole of the right parietal bone. A line circumscribing the base measured twelve inches. The child's health was at first undisturbed, but she was subsequently attacked with convulsions and died.

"On opening that tumour which was seated over the parietal protuberance, it was found to be filled with coagulated blood. The red particles had subsided for a depth of about three lines; the dark clot beneath was more than half an inch in thickness, firm in texture, and adhering closely to the bone; especially near that situation where the depression had existed between the two tumours. The other, more distinctly fluctuating tumour, contained semi-coagulated blood, of the appearance and consistance of gooseberry jelly, and somewhat less firmly adherent to the bone than was the clot in the other situation. After this blood had been removed, a semi-circular layer of dense, reddish, fibrinous exudation, about three lines broad, wedge-shaped, with its narrow edge directed inwards, was seen extending along the inner and anterior border of the tumour. It was situated immediately beneath the pericranium, adhered very closely to the subjacent bone, and formed, so far as it extended, a raised margin around the effused blood. The sensation it conveyed to the finger was very similar to that which is communicated by the osseous ring that usually surrounds these effusions, though its edge wanted something of the sharpness which that presents. The subjacent surface of the parietal bone was rough and uneven; a condition which, moreover, was not confined to that situation, but existed over a large extent of the bone. This roughness seemed to be owing to the deposit of new osseous matter, which apparently had formed the medium of the firm connection that existed between the clot and the surface of the skull." P. 401.

A fissure was noticed in the right parietal bone.

"On the inner surface of the bone was an effusion of blood between the cranium and dura mater, more than half an inch in thickness, and occupying the whole of the fossa of the parietal bone. The tumour thus formed was of an oval shape, and its surface was slightly irregular. Between the two layers of the dura mater, by which it was covered, were numerous bony deposits, and a ring of newly-formed bone surrounded its base. This ring had not attained the same development in its whole circumference; osseous matter having been abundantly poured out along the frontal margin of the tumour, more sparingly elsewhere, and being entirely absent along its inner border. It appeared to have been formed partly by ossification of the dura mater, partly by heaping up of new bone on the inner surface of the skull. It would have been impossible to ascertain whether the inner table of the skull had contributed, like the outer table, to the process of reparation, by the deposit of new bone, except by the complete removal of the clot, and consequent destruction of the preparation. The clot, therefore, was raised at one edge only, but in that situation the bone was perfectly smooth and unaltered. The blood appeared to have been poured out in successive layers, none of which, however seemed of very recent date, since all were firmly coagulated, though the red particles had not subsided, but remained equally diffused throughout the clot." P. 402.

There was a depression of the right hemisphere of the brain in a situation corresponding to the tumour; with this exception, there was no other appearance of importance.

The author remarks that, the two features which impart to this case its chief interest, are the fissure of the parietal bone, and the effusion of blood upon the dura mater, both of which have been but rarely noticed. In the absence of any proof of injury having been inflicted on the child, either before, during or after birth, it is not very easy to account for the fissure of the skull. The probabilities appear to be in favour of its occurrence during labour, of which accident there are several cases on record.

Dr. West does not regard the effusion of blood on the surface of the dura mater as merely the result of hæmorrhage from the vessels wounded by the fracture of the bone, but considers that the fissure and effusion probably occurred at the same time, and from a similar cause. The yielding structure of the infantile skull, with its membranous fontanelle and unossified sutures, is probably the reason why the effusion of blood upon the surface of the brain does not at first, nor invariably, cause cerebral symptoms.

The affection described in this paper is well know to practical accoucheurs, but we have no where met with so complete and satisfactory account of it as that given by Dr. West.

XVI. Large Opening into the Anterior Part of the Urethra caused by Sloughing, and attended by considerable Loss of Structure, successfully treated by Operation, with Remarks. By *F. Le Gros Clark*, Esq. Assistant Surgeon to St. Thomas's Hospital.

Mr. Clark, after noticing the difficulty of curing urinary fistulæ generally, but more especially those situated in that part of the urethra which is anterior to the scrotum, quotes the experience of Professor Dieffenbach, " who, having succeeded in healing an opening in the anterior portion of the urethra, of sufficient size to admit a large catheter, exultingly congratulates himself on having arrived at the solution of an enigma which had so long puzzled him, and on having mastered a difficulty which had so often foiled him." Dieffenbach attributes the failure of these operations to the presence of the urine, which it is next to impracticable to keep from the edges of a newly-pared wound communicating with the passage. But another difficulty, and one which peculiarly applies to the anterior portion of the urethra, is the extreme thinness of its wall, and tenuity of the integument in this position; and thus, when the edges are fairly brought into contact, there is not superficies enough to present a prospect of union by the first intention. " It has been remarked that the tendency of the *edges* of a wound to unite is proportioned to the extent of surfaces brought into contact; being feeble where the skin is thin, and comparatively vigorous where it is dense and richly supplied with vessels. This axiom being admitted, it is merely a further extension of it to assume that, where two *surfaces*, instead of edges, can be brought into relation with each other, the adhesive tendency would be proportionately great." This, which is the principle of Dieffenbach's operation, was adopted by the author in the treatment of the following case.

A wine-porter had permanent stricture, for which he was operated on by Mr. Clark; but, being of intemperate habits, and not very good constitution, he had acute inflammation of the testicle, followed by sloughing of the scrotum and penis, which, after the healing process was completed, left an aperture in the urethra one inch and a quarter in length, and occupying a small portion of the scrotal division of the passage, and extending forwards for a considerable distance anterior to it. The following were the steps of Mr. Clark's operation for the closure of this opening. A

small inverted portion of skin was first dissected out from the scrotal extremity of the fistula; four incisions were then made, two of which extended downwards and outwards over the scrotum, and the other two upwards and outwards on the sides of the penis; a lateral flap was thus marked out on each side, which was then dissected up; and the two being brought together, *surface to surface,* over the fistulous opening, were maintained in that position by lateral splints of leather, and sutures passed through the latter and skin. A semilunar incision was then made on each side, to relieve the anticipated tension when swelling came on. A full-sized gum-elastic catheter which had been passed prior to the commencement of the operation was left in the bladder. Adhesion took place, throughout the whole extent of the flaps, except at two points, one being at the scrotal extremity of the fistula, the other between the lateral supports: these were freely touched with caustic, and soon closed. A small portion of the margin of one flap sloughed. The operation was performed in November; and at the present date (June 9th) the patient continued to pass his water in a full and natural stream. The small perineal aperture still allowed of the occasional passage of a drop of urine. There is no contraction whatever of the penis.

This case is interesting as showing the practicability of the principles acted upon by Dieffenbach, and is creditable to Mr. Clark's surgical skill.

### XVII. The Pathology of Mental Diseases. By *John Webster,* M.D. F.R.S.

The present paper may be viewed as a continuation of that on the Statistics of Bethlem Hospital, from the pen of the same author, published in the 26th Volume of the Transactions of the Society. He enters, however, upon new matter, when he gives a table exhibiting the numbers of admissions, cures, and deaths occurring among the male and female patients in Bethlem Hospital during the last twenty-two years, and arranged according to the season in which they took place. In drawing the inference from this table that "as the temperature of the weather increased so did mental diseases become more frequent," he seems to have fallen into the error of confounding the times of admission in the several cases with the times at which the patients were first attacked by the disease. Now we have no data to show that these times were so nearly coincident as to furnish similar results in reference to the quarter of the year in which the grestest number of them would be found to have fallen if separately estimated; while the only fact which it concerns us to ascertain is the time of year, or state of weather most marked by the breaking out of the malady, not that different, and later time at which there may have been the greatest influx into an asylum.

The year being divided into quarters of three months in each, the first quarter consisting of the first three months and so on; it appears from the table that, during the second and third quarters of the 22 years referred to, the total number of curable lunatics admitted amounted to 2,736, whereas, during the first and fourth quarters, the number was only 2,239, being less than the former by 497. It is stated, moreover, in regard to particular months, that the greatest number of curable lunatics were admitted in May, the fewest in January.

Respecting the curability of insanity, it appears that only 986 were discharged convalescent in the first and second quarters taken together, whereas, during the third and fourth quarters, 1,569 patients left the institution free from mental disease. The numbers of deaths in the several quarters taken in order were 74, 55, 62 and 64 respectively.

A subsequent table gives us the gratifying intelligence, that the average weekly number of patients under restraint, amounting in 1840 to 13½, had been 9, 3 and 3 in the several three following years, and was no more than 1½ in the year 1844; thus encouraging the hope that, at no distant data, the whole system of coercion will become a dead letter at Bethlem, as it already has at Hanwell.

The most valuable part of the paper follows, viz. "a report of 36 dissections of insane patients recently performed at Bethlem Hospital, making, with the 72 contained in the previous paper, 108 autopsies," for an account of which the Society and the public are indebted to Dr. Webster. These descriptions state the sex, age, and time of residence in the hospital of the deceased, we regret that the whole duration of the mental malady, its general character, and the manner cf death have not been also briefly noticed, since the addition of these particulars would, we think, add much to the interest of the report, without greatly increasing the labour of the compiler. The morbid appearances themselves are concisely and clearly detailed, and are entitled to our confidence, as we have Mr. Lawrence's voucher for their accuracy. Of these, of course, we can give no detailed account, but shall content ourselves with quoting the summary appended by the author, which will convey in small compass a great deal of useful information.

"According to the above statement, some diseased alterations of structure, more or less evident, in the brain and membranes, were observed in all the thirty-six dissections now detailed; of which the following summary may be given: In thirty-three cases the pia mater was infiltrated. In thirty, there was turgidity of the blood-vessels of the brain and its membranes. In twenty-six, effusion of water had taken place in the ventricles. In sixteen, there was thickening and opacity of the arachnoid coat. In twelve, fluid was met with at the base of the brain. In nine, the consistence of the brain was altered from its normal condition. In eight, patches, or bloody points, appeared on the cut medullary surfaces. In five, the colour of the medullary, or cortical substance, was altered from its healthy hue to a pink, mottled, or rosy tint; and in four cases, blood was effused in the brain, besides other morbid changes of structure of a less important character; for an account of which I would refer to the synopsis, to avoid superfluous repetition. The same may be said in regard to the cases exhibiting diseased alterations of structure in the organs of the chest; of which description there were thirty examples; being five-sixths of the dissections contained in the present series; whilst only twelve patients, or one-third, showed any morbid appearances in the abdominal viscera.

"I subjoin a short abridgment of the pathological changes met with in the brain and membranes of the one hundred and eight autopsies reported in the present and previous communication to the Society. I may remark, that infiltration of the pia mater was observed in ninety-two cases. Turgidity of the blood-vessels existed in eighty-nine; fluid was effused in the ventricles in sixtyseven; effusion had taken place at the base of the brain in thirty-nine. There was thickening and opacity of the arachnoid coat in thirty-two. Bloody points were observed on the cut surfaces of the medullary substance in twenty-seven. The colour of the brain appeared changed in nineteen; and in seventeen cases, blood was effused within the cranium. These data indicate unequivocally, that the morbid alterations of structure, characteristic of insanity, which pathologists may expect to find in a majority of cases, will be, infiltration of the pia mater, turgidity of the blood-vessels, and effusion of fluid in the ventricles." P. 451.

We could wish Dr. Webster to be a little more particular in the language which he employs when giving numerical details, since he might thus avoid some obscurity, as well as some inaccuracies, as when he uses the term "relative proportion," or speaks of "a number as exceeding a ratio." We cannot promise always to adopt his inferences, but we are, and ever shall be, thankful to him for his facts, and of these we hope that he will not be chary in the supply.

### XVIII. On some of the Causes of Pericarditis. By *John Taylor*, M.D. &c.

At the outset, the author divides cases of pericarditis into two classes, viz. 1st. Those occurring in persons previously in good health, or in the course of some sthenic acute disease, as rheumatism; and 2nd, those occurring in persons previously in bad health, or in the progress of some chronic disease.

Of 25 cases subjected in the first instance to analysis he finds 15 to belong to the first and 10 to the second class; and the 15 (except that there is some little doubt about two of them) all occurred in the progress of acute rheumatism. Of the 10, 6 certainly and 1 probably, were accompanied by Bright's disease, and in 3, the state of the urine and kidneys was unknown. These cases might be expected to form the subjects of the immediately following observations; but it is not so in this lengthy and ill-digested paper, six other cases, which are submitted to a different principle of classification (being arranged under four divisions) are embodied with them in establishing the conclusion that pericarditis is of frequent occurrence in connection with acute rheumatism, and also with Bright's disease, while others are added as make-weights in a note, which are not brought into the calculation in the text.

Dr. Taylor next indulges in a theoretical discussion in order to make it appear "probable that the immediate cause of pericarditis and other internal inflammations which so often complicate Bright's disease and acute rheumatism, as well as of the articular affection itself in rheumatism, is a morbid condition of the blood;" and he says, farther on, that "there may be," in this latter disease, "an excess of urea, and perhaps also of other irritating principles in the blood." To us it appears idle to speculate upon a question which can only be determined by direct experiment, and which that may so readily set at rest.

### 2. *Of the causes of the pericarditis in cases of old adhesion of the pericardium.*

After a more particular account of 20 cases, in which old adhesions of the pericardium were discovered after death, we have an interesting summary in the following words :—

"Of the twenty cases of adhesion of the pericardium, therefore, there are *three only* in which the previous existence of pleurisy, or of rheumatism, or the *actual* or *probable* presence of Bright's disease, was not *ascertained*, and the *absence* of all these diseases was *ascertained* in *no one case*. With one exception, the adhesions of the pleuræ were either *double*, or on the *left* side; and in this exceptional case the adnesion of the pericardium was over the *right* auricle only." P. 465.

3. *Of the causes of the pericarditis in cases of white spots on the pericardium.*

Of 83 cases where white spots were found on the pericardium without adhesions, there were 58 in which there was found either Bright's disease or some other renal disease, or in which there had formerly existed acute or chronic rheumatism.

In 23 cases of the 83, there is no information given respecting either rheumatism or renal disease; and in 2 cases only was the absence of both these maladies ascertained, and in these pleuritic adhesions existed.

Of these 23 cases, there were 11 with old adhesions of the pleuræ, 6 in which there were none, 6 in which this fact was undetermined.

Of 22 cases of the 83 with white patch, without previous rheumatism but with some disease of the kidneys, 17 had old adhesions of the pleura. Dr. Taylor concludes, from the examination of these cases,

1st. That in all of them there *may* have been either rheumatism, or Bright's disease, or inflammation of the pleura.

2dly. That in 17 cases only the existence of any of these diseases was not ascertained, and he thinks that he has shown, though we cannot go with him quite so far, that one or other of the three causes under discussion was ascertained to exist in almost every case examined.

Of the re-capitulated reasons for believing pleuritis to be a cause of pericarditis we need only mention one, namely, that in one class of cases where the two diseases co-exist, the post-mortem appearances show the disease in the pleura to be older than that in the pericardium.

We pass by the statement of the opinions of other authors, viz. Messrs. Louis, Bouillaud and Hache, on the causes of pericarditis in general, of those of Drs. Hope, Watson, and Williams, on rheumatism as one of them, and of those of Drs. Bright, Christison, Rilliet and Barthez, on albuminuria as another, and we give the comparative results in Dr. Taylor's words :

"The chief particulars, therefore, in which my results differ from those obtained by other observers, are these :—1. I have found an adequate cause for the pericarditis in *all* my cases. 2. Whilst I have found rheumatism, as a complication, in quite as great a proportion of cases as any one who has given us a numerical account of his observations, yet its frequency, relatively to other causes, has been less in my experience than in that of any writer with whom I am acquainted. 3. I have found Bright's disease to be the cause of pericarditis in a large proportion of the cases which have fallen under my notice." P. 478.

The next head of investigation is

I. OF RHEUMATISM CONSIDERED AS A CAUSE OF PERICARDITIS.

1. *Frequency with which pericarditis and endocarditis occur in rheumatism.* —In an analysis of 47 cases of acute or subacute rheumatism that occurred in the practice of Drs. Elliotson, Carswell, and Williams, we find 11 instances of valvular disease probably old in most of them, 2 of slight recent valvular disease, and no evidence of any disease in the rest.

Again, in 86 cases, of the same kind, were found 5 of acute pericarditis, 19 of probably for the most part old valvular disease; and, respecting the rest, in 19 there was no evidence of heart-disease, and in 43, none concerning the condition of the heart.

In another set of 75 cases, treated by Dr. Taylor himself, 8 had pericarditis, and in 2 more it was suspected, while there was valvular disease, old or recent, in 34, in 4 of which pericarditis also existed.

The published experiences of Bouillaud, Hope, Williams, Macleod, W. Budd, Rilliet and Barthez, and Chomel, on the question of the frequency of endocarditis and pericarditis in rheumatism, are passed in review, and exhibit very considerable discrepancies. Thus it is stated at page 490, "If we take pericarditis and endocarditis *together*, we shall find that Bouillaud observed this complication in $\frac{6}{7}$ of the cases of acute rheumatism, Dr. Williams in fully $\frac{3}{4}$, Chomel in about $\frac{1}{2}$, Dr. Hope in $\frac{1}{2}$, and Dr. Budd in $\frac{1}{2}$. Dr. Taylor found the same complication in $\frac{1}{2}$ cases treated by himself, and in $\frac{2}{7}$ of those treated by the other physicians to the hospital, conjointly."

In the cases of acute rheumatism occurring in University College Hospital, there was found to be *recent* endocarditis or pericarditis in 1 in 17 of those treated by Drs. Carswell and Williams; in 1 in 5, about, of those treated by Dr. Taylor himself.

He thinks that this greater proportion of recent heart-affections in his own cases, as compared with those of his colleagues and of M. Chomel, arises from his having observed and recorded some slight cases which others might have thought unworthy of note; while he conceives that the smaller proportion of instances of *recent* pericarditis and endocarditis in his own practice as compared with the results obtained by many other physicians, is due to their classing together all the cases in which valvular murmurs were first observed in connection with rheumatism, and not separating the cases of *recent* from those of old affections of the heart, of which last he makes it appear probable that the proportion was very considerable.

Dr. Taylor states, in a note, that having examined with care 1,026 patients labouring under every variety of disease on their first admission into University College Hospital, he found a murmur indicative of valvular disease of the heart in 385 cases, that is in 37·524 per cent. But of these patients there were 82 suffering from acute or sub-acute rheumatism, of which 82 cases 55 were accompanied by cardiac murmur. Excluding then these cases, we have 944 persons suffering under all kinds of disease, except rheumatism, of whom 330, that is nearly 35 per cent., have cardiac murmur.

Dr. Taylor then recurs to his 75 cases of rheumatism, mentioned sometime before, in 34 of which, valvular murmurs were heard, and derives from this series of observations the ratio of 45·33 per cent. as a standard whereby to measure the frequency of cardiac murmurs in cases of acute rheumatism generally.

He then proceeds to say "The *excess* in the amount of valvular disease in cases of acute rheumatism, therefore, above that in *all diseases indiscriminately*, is 7·809 per cent; and above that in *all diseases exclusive of rheumatism*, 10·33 per cent."

Now if Dr. Taylor, instead of taking the 75 cases of acute rheumatism exhibiting 34 of valvular disease from which he obtained the standard measure of 45·33 per cent., had taken (and why should he not have done so) the 82 cases of acute and sub-acute rheumatism mentioned in this note, in 55 of which valvular disease was found to exist, he would have obtained 67 per cent. as his standard, and the two excesses would have been 29·476 per cent. and 32 per cent. respectively, or more than three times as great as in the former case. Thus we see how entirely numerical results depend upon the data from which we start, and that conclusions deduced by means of arithmetical calculation, though arrived at by a succession of steps of which many are perfectly trustworthy, partake nevertheless of the imperfection, and insecurity of the grounds upon which they are based.

Our author inclines to the belief that the excessive proportion of valvular murmurs observed by Bouillaud must be owing to his reckoning the inorganic murmurs occurring in patients who were either anæmic at the commencement, or rendered so by his treatment. He next proceeds to another branch of his subject.

"If we consider *acute pericarditis* apart from endocarditis, or valvular disease, the results are as follows. Bouillaud found this complication in one-half of his cases of acute rheumatism. Dr. Macleod, in one-fifth in *adults*, and in one-half in children (four in eight.) Rilliet, in one-third in children. Dr. W. Budd found it in one out of every eight and a half cases; and I found it in one out of twelve and a half of *my own* cases, (or, including two *slight* cases, in one in nine and a half,) and in one out of seventeen of the rest of the cases observed in University College Hospital." P. 499.

Upon these statements he remarks that, for reasons which we omit, Dr. W. Budd and himself alone give data which admit of fair comparison, and the results are very nearly the same; and the general inferences from the preceding investigation are stated to be—

"1. That acute inflammation of the heart has occurred less frequently as a complication of rheumatism in Dr. Taylor's experience than it has been *believed* to occur in the experience of those writers whose opinions seem to have been most generally adopted by the profession."

"2. That the frequency of inflammation of the heart even in Dr. Taylor's cases has been such as abundantly to show the great influence of acute rheumatism in its production."

2. *Frequency of Morbus Cordis in* CHRONIC *Rheumatism.* Out of 109 cases of chronic rheumatism *taken indiscriminately*, 20 are stated to have had some disease of the heart, chiefly valvular, two only had acute pericarditis, and 87 had no disease of the heart.

"If we compare," says Dr. Taylor, "these cases of chronic with the eighty-six cases of acute rheumatism, which were observed under the same circumstances we find,—

"1. That the proportion of cases of disease of the heart, *chiefly* valvular, and believed to be *chiefly* old, is nearly the same in both classes, being about 22·09 per cent. in the cases of acute rheumatism, and about 18·34 per cent. in those of the chronic form.

"2. That there is however a small excess of the variety of cases of morbus cordis under consideration among the cases of acute rheumatism, which is probably due to a greater proportion of examples of *recent endocarditis* in this form of the disease.

"3. That the *amount* of the excess just referred to is 3·75 per cent., and assuming it to depend wholly upon *recent* endocarditis, these figures will represent the *amount* of the excess in the proportion of cases of *recent* endocarditis in acute rheumatism *above* the proportion in chronic rheumatism." P. 505.

3. *Frequency of other internal Inflammations than those of the Heart in* Acute *Rheumatism.*—Some details are given under this head which it is needless to repeat, as the author himself says that he does not attach much importance to the results which they furnish. They tend to show that inflammations of the lungs, pleura, and parts within the head, are next in order to those of the heart, and that those in the abdomen are the least frequent. An examination of the same point, in reference to chronic rheumatism, gives no more satisfactory information.

Under the head 5, *Of the Circumstances which favour the occurrence of Pericarditis in the course of Rheumatism,* we have metastasis dismissed, we believe very justly, "as not a *frequent* cause of pericarditis." This conclusion is corroborated by a reference to the experience of Drs. Watson, Hope, M. Hache, and Dr. Graves; and the supposed occurrence of rheumatic fever without arthritis, in which the heart has become affected, favours the same view.

As regards the form of rheumatism, if we adopt the division of febrile rheumatism into a fibrous and capsular variety, all the cases of rheumatic pericarditis were observed by Dr. Taylor, and almost all of them by Dr. Macleod, to arise in the former of these.

Something like a presumption is created in favour of the truth of the following inference, viz. that the violence and fatality of rheumatic pericarditis are *generally* greater in the cases in which the rheumatism is *very* acute, than in those in which it is subacute. Whether pericarditis is more *frequent* in the very severe, than in the less severe form of acute rheumatism, is uncertain; neither do we find any guide as to the stage of the rheumatism, in which the pericarditis is wont most frequently to commence. But there seems good reason to believe that rheumatic pericarditis is more frequent and more severe in the first than in subsequent attacks of rheumatism. Previous disease of the heart probably does not increase the liability to attacks of pericarditis in rheumatism. Of the age and sex most liable to this disease, nothing is added to our previous knowledge. Neither can we place any reliance on the observations in reference to the influence of venæsection; we quite agree with the author, that the facts adduced do not meet the real question, and we therefore wonder at his citing them. Plausible hypotheses are too abundant for us to stop to examine Dr. Taylor's, on the mode in which rheumatism produces pericarditis.

II. OF BRIGHT'S DISEASE OF THE KIDNEYS, AS A CAUSE OF PERICARDITIS, AND (INCIDENTALLY CONSIDERED) OF SOME OTHER INFLAMMATIONS.

In 31 cases of pericarditis observed by Dr. Taylor, 9, if not 11, had Bright's disease. To determine the frequency of pericarditis, and also of some other internal inflammations in cases of Bright's disease, the author next details the number of internal inflammations, under seven heads, either actually existing, or leaving traces of their previous existence, in 50 patients who laboured under this disease, and whose bodies he had examined after death.

The same process being applied to 100 similar cases of Dr. Bright's, published in the Guy's Hospital Reports, numerical results are obtained differing so widely from the former as to show, not that either series of observations is defective or unworthy of credit, but that no calculation of average results can securely be founded on either of them taken alone. And this conclusion is still farther confirmed by the results of an analysis of 129 cases of M. Becquerel, and of 33 of M. Malmsten's, differing considerably from each other, and from those of each of the two series just spoken of. Yet it is from his own 50 cases alone that the author draws his subsequent reasonings.

Dr. Taylor next seeks to determine the frequency of pericarditis, and of some other inflammations, in patients without Bright's disease, by a reference to 142 post-mortem examinations of patients whose kidneys were healthy. We give the results in his own words.

"(1.) 50 cases of renal disease yielded 48 examples of *acute* inflammations,—i. e. 96 inflammations in every 100 patients; and that these all occurred among 30 out of the 50 patients,—i. e. 60 per cent. of the cases were complicated with internal inflammations, and the number of these inflammations averaged more than 1½ to each patient affected. Also,

"(2.) 50 cases of renal disease yielded 81 examples of *previous* inflammations,—i. e. 162 inflammations in every 100 patients; and that these all occurred among 45 out of the 50 patients,—i. e. 90 per cent. of the cases were complicated with some previous inflammation, and the number of these inflammations averaged above 1¾ to each patient affected.

"On the other hand we find:—

"(1.) 142 cases *without* renal disease yielded 68 examples of acute inflammations,—i. e. 42 inflammations in every 100 patients; and that these all occurred among 51 out of the 142 patients,—i. e. not quite 36 per cent. of the patients had some acute inflammation, and the number of these inflammations averaged 1⅓ each patient affected. Also,

"(2.) 142 cases *without* renal disease yielded 179 examples of *previous* inflammations,—i. e. 126 inflammations in every 100 patients; and that these all occurred among 100 out of the 142 patients,—i. e. about 70½ per cent. of the cases were complicated with some previous inflammation, and the number of these inflammations averaged nearly 1½ to each patient affected." P. 543.

It has been seen, in a series of 31 cases of pericarditis mentioned at the commencement of this article, that 18 occurred simultaneously with acute rheumatism, and 9 co-existed with Bright's disease of the kidney, and this, as far as the number justify, a general inference indicates that rheumatism is twice as fertile in pericarditis as granular kidney. But this may be due to the fact of acute rheumatism being a malady of more frequent occurrence than Bright's disease, and the author wishes to know the comparative efficacy of the two in producing this affection of the heart. In relation to this enquiry, therefore, it becomes necessary to ascertain the relative frequency among physicians' cases of acute rheumatism and Bright's disease, and Dr. Taylor, though he cites facts which have no bearing on the question, yet furnishes sufficient data for the probable conclusion that $1\frac{1}{2}$ to 1 expresses nearly the relative frequency of these two diseases, and would make 4 to 3, on the grounds above stated, express the comparative efficacy sought for. Dr. Taylor, however, makes no application of the indefinite conclusion which he arrives at as to the frequency of the occurrence of the two diseases; but starts on a new tack in order to arrive more directly at the end proposed. Thus, finding 8 cases of acute pericarditis in 75 cases of rheumatic fever, and 16 of this disorder in 183 cases of Bright's disease, he draws the erroneous conclusion that Bright's disease and acute rheumatism appear to have caused acute pericarditis in an equal number of cases; whereas, the premises on which he argues would show the relative frequency of acute pericarditis in equal numbers of cases of acute rheumatism and Bright's disease to be as 11.4 to 9.375.

The conclusion that adhesions of the pericardium (and therefore *previous* attacks of pericarditis) have been produced much more frequently by Bright's disease than by rheumatism, appears to us supported by no sufficient evidence.

Some farther calculations of the frequency of acute endocarditis in the two diseases follow, and also of that of valvular disease, old and recent; with the conclusion that, in both instances, the heart-disease occurs with equal frequency in the two maladies. A kind of arithmetical resumé and farther calculations then follow, for which we must refer to the original paper; wherein will be seen the inconsistency of the results, as well as the explanation of the inconsistency. To the paper too we must refer for the statement of the relative frequency of other acute inflammations, and for the notice of the occurrence of pericarditis in other blood diseases. The author concludes by calling attention to the constitutional causes of inflammation, and expresses his opinion, " that, in very many cases of acute rheumatism, no undue exposure to cold or wet, or to the influence of any other (if there *be* any other) acknowledged exciting cause of the disease, can be ascertained to have preceded the attack within such a period of time as could justify us in regarding the two events in the relation of cause and effect.

In concluding our very imperfect sketch of this long and rambling paper, it would be great injustice to the author were we not to express our sense of the value of many of the facts, both original and others, which he has brought to bear upon some interesting pathological questions. We have to thank him more especially for the detail of several series of cases observed by himself apparently with much care and accuracy. We think that he has done good service, too, in calling attention to pleurisy as a sourceof pericardial inflammation, to Bright's disease, as a more influential agent than we had suspected it to be, in producing that and other internal inflammations, and to the fact of the endocardial murmurs first observed in rheumatic fever, having in many cases an existence prior to, and quite independent of, that disease. The paper seems to us highly useful as a suggestive one, if we may employ such a term; but we certainly can place little or no reliance on the results of the numerical calculation. There is evidence of great haste, and of but little pains, in its composition, considering the scope and bulk of this essay. Dr. Taylor seems throughout to have fallen into one great error; instead of sifting the materials at his command, rejecting what was imperfect or doubtful, but selecting and combining all that was trustworthy to form the basis of his calculations, he has founded these on less extensive data, and has consequently obtained only partial, and sometimes incongruous results, which he wastes much time in endeavouring to reconcile. On the whole, we think he would have done well if he had laboured, prior to its publication, to render his paper more concise and clear, if he had limited his enquiries to a smaller compass, but striven to make all the ground sure over which he passed, if he had omitted all facts but such as he had occasion to make use of, and if he had rigidly abstained from all hypothesis. As the paper stands, it is so long and crude that we almost wonder that space was given it among the Transactions of the Society.

XIX. Case of Excision of the Upper End of the Femur, in an example of Morbus Coxarius. By *William Fergusson*, Esq. Professor of Surgery in King's College, London.

The subject of this operation was John Clark, æt. 14, a patient in Kings's College Hospital. The condition of the diseased limb is thus described by Mr. Fergusson.

"The shaft of the femur sloped obliquely downwards and inwards, and the knee rested on the inner side of the thigh of the sound limb; the head of the bone could be felt, through the soft parts, lying on the dorsum ilii, and its identity could be more accurately ascertained by passing the finger into a large sinus which opened on the surface over and behind the trochanter major. The articular extremity was so isolated that the finger could be passed round it in all directions. There were several small sinuses contiguous to the large one, but it could not be ascertained that any of them led to diseased bone, or communicated in any way with the pelvis. There was a large circular sore which occupied the whole of the skin over the trochanter major, and profuse discharge of thin matter from the open surfaces." P. 572.

As the disease appeared likely to prove fatal, and as the circumstances of the case seemed peculiarly eligible for excision of the head of the femur, the author, with the concurrence of his colleagues, determined to have recourse to the operation.

"A longitudinal opening about six inches long was made in the line of the femur, extending from over the head of the bone to a little below the trochanter major, and the tissues were separated from the shaft of the bone, a little below that process, so as to permit a curved needle to be used for the introduction of a chain saw. This latter step was attended with considerable difficulty owing to the depth and obliquity of the bone, and when accomplished proved of little value; for after several trials the instruments (which worked very indifferently in my hands) broke, and I was compelled to adopt another mode of procedure. With a sharp-pointed bistoury I separated all the soft parts from the neck of the bone and the trochanters, and then, by causing the knee to be moved across the opposite thigh, and using the femur as a lever, the head and portion of the bone thus isolated was so thrust out of the wound that I could with facility apply the ordinary saw for the requisite section. Not being satisfied with the condition of the interior of the bone at the surface exposed by the saw, I enlarged the opening, and removed about three quarters of an inch more, then closed the wound with a few points of interrupted suture, and covered it loosely with a pledget of lint. No vessel of sufficient magnitude to require a ligature was divided. The cotyloid cavity was filled by a fibro-gelatinous mass, similar to the lining of the sinus." P. 574.

The effects of the operation were not severe. Part of the wound united by the first intention. The patient improved in strength, and six months after the operation, it is stated, that the boy walks about on crutches, and, besides looking hale and plump, expresses himself as being in good health. There is free movement both at the knee and hip, and the sinuses have all but closed.

This case, so far as the author can learn, is the second of the kind attended with a favourable result which has occurred in Britain. The first case occurred to Mr. Anthony White in the year 1818, in the Westminster Hospital. Sir Benjamin Brodie has also removed the head of the femur, and a similar operation was performed in Dublin by the late Mr. Hewson.

"In both these instances the patients died, but the circumstances were not so favourable as in Mr. White's case and my own, for the parties were adults; and in Sir B. Brodie's, the head of the bone was in the acetabulum at the time of the operation. This patient died within a few days after, apparently the direct effect of that proceeding. Mr. Hewson's died three months after, from profuse suppuration, and the cotyloid cavity after death was found to be carious." P. 577.

These are the only examples in which the operation has been performed by British surgeons. Bourgery gives the names of ten surgeons in other parts of the world who have also performed it; of these patients, one-half seem to have died from the proceeding.

Without questioning the propriety of the operation in the preceding case, we consider the excision of the head of the femur to be very rarely applicable, and certainly our present experience of the results of the operation is far from being encouraging. It appears to be adapted to those cases in which the carious head of the bone is displaced upon the dorsum ilii, as in Mr. Fergusson's patient, and keeping up an exhausting discharge from the neighbouring sinuses, whilst the cotyloid cavity is free from disease. Under such circumstances, the bone being but thinly covered and no vessels interfering, the operation would be neither difficult nor formidable.

XX. On the Minute Structure of the Lungs, and on the Formation of Pulmonary Tubercle. By *George Rainey*, Esq. M.R.C.S. Microscopist of St. Thomas's Hospital.

In this paper, Mr. Rainey gives the results of his investigations on the structure, functions, and pathology of the lungs with the aid of the microscope. His observations on these subjects, especially on the formation of tubercle, are highly interesting, and are evidently founded on a careful examination of the diseased organ. We regret that these researches cannot be presented to our readers in an abridged form, and that we must therefore be content with this expression of our favorable opinion of the author's labours.

XXI. Two Cases of Aneurism in which there was neither Pulsation nor Abnormal Sound. By *T. A. Barker*, M.D. Physician to St. Thomas's Hospital.

The author observes, that no medical man can have been long in practice without meeting with cases in which extensive disease of the heart and large arteries had long existed, and ultimately proved fatal, without having caused any symptom directing the attention of the patient to the seat of disease; and that there must be few experienced practitioners who, when consulted for some other complaint, have not detected organic disease of the heart, or large vessels, which had previously been overlooked by some medical attendant, not because he was incapable of detecting such disease if he had looked for it, but because the patient had spoken of no symptoms referable to disease of those parts. Dr. Barker gives a minute history of two interesting cases of aneurism. In one case, a large aneurism of the arch of the aorta was not discovered until after death. In the other, an aneurism of the renal artery was not detected until the day before death. He remarks, that what makes these cases worthy of record, is, not that the aneurisms existed without giving rise to prominent and characteristic symptoms, but that, when certain symptoms had arrested his attention, leading him, in the first case, to think it highly probable, and, in the second, possible, that an aneurism existed; the most careful and frequently repeated examinations did not enable him to detect either pulsation or abnormal sounds. Dr. Barker states:—

"There have been other cases less frequent than the above, in which, after careful and repeated examination, no symptoms of aneurism were observed; but on examination after death, some cause for the absence of such symptoms was found, either in the size, shape, or situation of the aneurism, in the mode by which it was covered by other parts, or in the nature of its communication with the artery. In neither of the cases just related, can any such explanation be given, and the care and frequency with which the examinations were made, leave no reason to suppose that any material symptoms which could have assisted me in the diagnosis were present, and overlooked. They may therefore be regarded as aneurisms of considerable size, in situations where such disease can ordinarily be detected without difficulty, but which gave rise to none of the ordinary symptoms; and looking at them in this light, I think they are not unimportant additions to our stores of pathological knowledge." P. 609.

The publication of these two valuable cases reflects credit on Dr. Barker's candour, and shows an honest desire for the advancement of medical knowledge.

XXII. An Account of a Singular Case in which there was a Black Secretion from the Skin of the Forehead, and Upper Part of the Face. By *William Teevan*, Esq. M.R.C.S.

The patient was a young lady, aged 15, who was afflicted with a singular black discoloration of the forehead and upper part of the face. "The discoloration was first observed about the middle of last January, on the left under eyelid, near the internal angle of the eye; appearing at the commencement as a brownish spot, which in the course of four or five days assumed a jet black colour, and, gradually extended to the entire forehead and eyelids of both eyes. The discoloration never appeared on any other part of the body, and on the forehead was accurately limited by the hair. The patient stated, that on her attempting to wash the black matter off, it caused her so much pain, owing to the sensitiveness of the skin, that she desisted," and, until it was removed by the application of soap and water, she was not aware that it could be removed by ordinary means. The quantity of matter removed was sufficient to darken four basins of water as black as Indian ink. With the exception of occasional headache and pain in the chest the lady appeared in good health; the bowels were generally confined, the catamenia regular, but scanty; her complexion fair, and the hair of a light brown colour. Suspicion of imposition naturally enough arose, and care was taken to sift the case. On the 31st of March, Mr. Teevan

"Washed the black matter from the right under eyelid, at one o'clock, and never allowed her to leave my presence until five o'clock, up to which hour no discoloration or alteration in the appearance of the affected part had occurred. Being now, more than ever satisfied, that the patient was imposing, I left her in the drawing-room, and proceeded as far as the door, with the intention of returning home, when her father requested me to remain until seven or eight o'clock, assuring me, that previous to that hour I should be perfectly satisfied respecting the truth of his statement, as the discoloration, according to his observations, did not appear sooner than from four to six hours after ablution. In compliance with this request, I returned to my patient, who, during the few minutes I was absent, had not left the drawing-room. Miss ——— told me that she now began to feel a pricking and burning heat in the affected parts, which invariably preceded, from about a quarter to half an hour, the appearance of the discoloration, and that she was confident the blackness would very soon show itself. At half-past five o'clock, I was astonished at seeing a small dark spot appear upon the right under eyelid, near the inner angle of the eye, which kept gradually extending towards the right temple, so that, in half an hour from its first appearance, the eyelid had become quite black, the forehead, at the same time, assuming the same intense discoloration. I remained with the patiênt until eight o'clock, and felt perfectly satisfied respecting the truth of the alleged phenomena." P. 614.

On the 26th of April, the case was closely watched by the author and Dr. Hodgkin, and with the same result as on the 31st of March. On the 4th of May, the patient vomited two large basins-full of fluid, containing an immense quantity of black matter, which subsided to the bottom of the vessels. This black matter, when examined under the microscope, as well as by analysis, was found to be identical with the black matter taken from the face. The black matter continued up to the 23rd of May, when it entirely ceased. Neither internal nor external treatment had the smallest effect in checking the secretion. Dr. Rees found carbon to be the colouring principle.

Mr. Teevan states that, he believes there is no case on record similar to the present one, and he considers the secretion to be analogous to what occurs in melanosis. The cessation of the secretion of the black matter by the cutaneous exhalants of the forehead during the period it was ejected from the stomach, bowels and kidneys, although singular, is not unaccountable; the latter organs probably becoming a substitute for the former. The secretion was always more abundant during the night than the day.

From the unusual character of the above case, and the doubts entertained by some persons as to its genuineness, Dr. Cursham was desired by the Council of the Society to ascertain further particulars from Dr. Read of Belfast, who saw the case at its commencement, and also from Dr. Hodgkin. Both these gentlemen express themselves perfectly satisfied of the genuineness of the case. The Council were certainly right in instituting these inquiries, for the deceptions practised by young women, evince so much cunning and are so singular that, without strong and respectable testimony, the profession would probly have received the report of this case with doubt, and have questioned the propriety of one so suspicious being published in the Transactions.

